# Texture Analysis of Dried Droplets for the Quality Control of Medicines

**DOI:** 10.3390/s21124048

**Published:** 2021-06-11

**Authors:** Yojana J. P. Carreón, Orlando Díaz-Hernández, Gerardo J. Escalera Santos, Ivan Cipriano-Urbano, Francisco J. Solorio-Ordaz, Jorge González-Gutiérrez, Roberto Zenit

**Affiliations:** 1 Facultad de Ciencias en Física y Matemáticas, Universidad Autónoma de Chiapas, Tuxtla Gutiérrez, Chiapas 29050, Mexico; yolanda.carreon@unach.mx (Y.J.P.C.); orlando.diaz@unach.mx (O.D.-H.); gerardo.escalera@unach.mx (G.J.E.S.); 2 Instituto de Ciencias Aplicadas y Tecnología, Universidad Nacional Autónoma de México, Avenida Universidad 3000, México D.F. 04510, Mexico; 3 Escuela de Medicina, Universidad Autónoma de Coahuila, Piedras Negras, Coahuila 26090, Mexico; ecipriano@uadec.edu.mx; 4 Departamento de Termofluidos, Facultad de Ingeniería, Universidad Nacional Autónoma de México, Avenida Universidad 3000, México D.F. 04510, Mexico; fjso@unam.mx; 5 Center for Fluid Mechanics, School of Engineering, Brown University, Providence, RI 02912, USA

**Keywords:** quality control, medicines texture analysis, dried droplets

## Abstract

The quality control of medicines guarantees the effectiveness of treatments for diseases. We explore the use of texture analysis of patterns in dried droplets as a tool to readily detect both impurities and changes in drug concentration. Four types of medicines associated with different routes of administration were analyzed: Methotrexate, Ciprofloxacin, Clonazepam, and Budesonide. We use NaCl and a hot substrate at 63 ${}^{\circ }$C to promote aggregate formation and to reduce droplet drying time. Depending on the medicine, optical microscopy reveals different complex aggregates such as circular to oval splatters, fern-like islands, crown shapes, crown needle-like and bump-like patterns as well as dendritic branched and star-like crystals. We use some physical features of the stains (as the stain diameter and superficial area) and gray level co-occurrence matrix (GLCM) to characterize patterns of dried droplets. Finally, we show that structural analysis of stains can achieve 95% accuracy in identifying medicines with 30% water dilution, while it achieves 99% accuracy in detecting drugs with 10% other substances.

## 1. Introduction

Traditionally, the quality control of medicines is carried out by using near-infrared (NIR) spectroscopy [1,2], Raman spectroscopy [3,4], chromatography [5,6,7] and X-ray fluorescence (XRF) spectrometry [8], among many others. It is important to note that all these techniques are very effective but involve the use of sophisticated instrumentation and highly trained personnel, requiring careful and controlled conditions.

In this investigation, we propose using the pattern left by an evaporated drop as a unique signature of each compound, unaltered or not. The analysis of patterns formed by droplet evaporation has already been used in many fields, such as diagnosis of pathologies [9,10,11], bioassays analysis, study of bacteria motility [12,13,14], detection of structural changes in liposomes [15], development of agricultural products [16,17,18], among many others [19,20,21,22,23,24,25]. One of the main objectives of the drop evaporation method is to create, in dried residues of droplets, certain morphological characteristics that may be easily identified and quantified. For example, a small fractal-like structure in the center of a uniform deposition reveals the coexistence of two different proteins [26]. The “coffee ring effect” as a Low-Cost tool has been used for the detection of protein mutation [27] and the estimation of β-sheet content of Human Serum Albumin [28], adenovirus in human tears [29], malaria [30,31], antibiotics [32,33], among many other components.

Particularly, residue patterns in saline droplets exhibit an enormous diversity of structures, as opposed to the case of drops without salts. Multifractal growth of crystalline aggregates emerge in the saline aqueous medium of gelatin [34] and liposomes [35]. Salts and proteins show an enormous diversity of aggregates such as amorphous aggregates, scallops, zigzag patterns and dendritic shapes [36,37]. Moreover, different aggregates emerge from the interaction among different salts and proteins [38].

The morphological difference among patterns resides in the high sensitivity of the salt crystals to be affected by control parameters such as the kind of salt, concentration, or the rate of evaporation. Therefore, some groups have used salt crystals as an indicator of the chemical and biological state of the components of a solution. Spermatozoa motility is scrutinized by the formation of elongated crystals with lateral tips that interlock with small salt aggregates [14]. In order to classify and differentiate alcoholic drinks and their possible adulteration, NaCl can be used to induce the formation of complex aggregates [39]. The premature rupture of membranes and/or the onset of labor can be diagnosed by observing a ferning pattern in dry vaginal secretions. This is the so-called “Fern test”, which is based on the presence of sodium chloride in mucus under the estrogen effect [40]. The presence of sodium chloride in blood serums deposits can be used as an indication to detect viral hepatitis in patients [41].

The morphology of a stain is architectured by two competing processes: the flow inside the drop, induced by the mass transport during evaporation, and the complex aggregation processes of the compounds in the liquid. A common circular structure, like a “ring”, is the vestige of the intense colloid transport from the droplet center to its edge. This effect, the so-called “coffee ring effect”, takes place due to the loss of water molecules is mostly stronger at the contact line, which induces a capillary flow radially outwards [42,43,44]. The complete transport of mass in the interior of a droplet depends on the competition between capillary flows and Marangoni flows [45]. Marangoni flows can prevent the coffee ring formation [46,47,48]. Surfactant and temperature gradients are responsible for forcing the fluid to redistribute into the droplet. Interestingly, the evaporation-induced flow inside the drop is practically the same for ultrapure water and mineral water with low content of minerals as that of water, but changes at high content of minerals [49]. The formation of more complex aggregates accompanies the slowing down of the strength of the internal flow due to the presence of ionic concentration gradients. The aggregation phenomena between colloid-colloid and colloid-substrate depend on the balance among friction force $F_{drag}$, the electrostatic force $F_{el}$ and adhesion force $F_{ad}$ by the interaction between the macromolecules and the glass substrate [44,50].

In this paper, we propose to apply texture analysis of dried droplets patterns as a tool to the quality control of medicines. We evaluated four types of commercial medicines associated with different routes of administration: Methotrexate, Ciprofloxacin, Clonazepam, and Budesonide. The analysis was carried out by mixing tap water, and other substances, with the drugs. Pattern formation of stains was induced by favoring ionic interactions, via the addition of salts, to induce crystal formation; and by increasing temperature substrate to dry droplets at a faster rate. This strategy allowed us to form, in a shorter time, patterns in dried droplets that can be used to identify medicines. Using standard optical microscopy, we found a great diversity of structures such as circular to oval splatters, fern-like islands, crown shapes, patterns of crown needle-like and bump-like as well as dendritic branched and star-like crystals.

We will show that patterns of modified drugs with water are sufficiently differentiated from the unaltered compounds by characterizing some physical features (as the stain diameter and superficial area) and using the gray level co-occurrence matrix (GLCM). We will demonstrate that, depending on the drug, this method detects 10% other substances (regular tap water, alcohols, consumable liquids, and active substances), with accuracy from 72 up to 99%.

## 2. Materials and Methods

### 2.1. Solution Preparation

Four medicines were used: Methotrexate (50 mg/2mL), Ciprofloxacin (3 mg/mL), Clonazepam (2.5 mg/mL) and Budesonide (1.28 mg/mL). To induce pattern formation in dried droplets, the drug stock solution contained 5% of a NaCl solution (at 10 wt%) and 95% of the sample of medicine.

These concentrated solutions (5% of NaCl and 95% of medicine sample) were diluted with regular tap water in varying amounts to the desired concentrations from 10% to 90%. Methotrexate solutions with other substances were prepared by mixing the Methotrexate stock solution and 10% of a consumable liquid (ethanol, methanol, isopropanol, rehydrating serum, carbonated drink and liquid detergent). We also used Folic acid, Diclofenac, and Acetaminophen as impurities. These drugs were dissolved in water (25 mg/mL). Methotrexate solutions with impurities were prepared by mixing the Methotrexate stock solution and 10% of these solutions of drugs.

Manufacturers of medicines are indicated as follows: Methotrexate, manufactured by Accord Healthcare Ltd. Clonazepam, marketed under the trade name Rivotril by Roche. Ciprofloxacin manufactured by PiSA laboratories. Budesonide, marketed under the trade name Rhinocort by AstraZeneca.

Additionally, we used 10 drugs to show that different medicines generate different patterns in dried droplets (one Proton-pump inhibitor, one antifungal, four antibiotics, four Non-steroidal anti-inflammatory drugs). Drug powders were dissolved in deionized water (Millipore, 18.2 MΩ cm), to reach 1.25 mg/mL. The drug stock solution was prepared by mixing 5% of a NaCl solution (at 10 wt%) and 95% of medicine solution. Manufacturers of medicines are indicated as follows: Pantoprazole marketed under the trade name Efectreflu by Apotex Inc. Fluconazole marketed under the trade name Ameztram by Sahuayo Raam Laboratory. Clindamycin marketed under the trade name Indacil capsule by Maver Laboratories. Nitrofurantoin marketed under the trade name Macrofurin by Mavi Pharmaceutical. Cefaclor marketed under the trade name Fasiclor capsule by Maver Laboratories. Metronidazole-Diiodohydroxyquinoline marketed under the trade name Flagosil 400 by Collins Laboratory. Meloxicam-Methocarbamol marketed under the trade name Flexiver by Maver Laboratories. Naproxen-Carisoprodol marketed under the trade name CARIDOXEN by Mavi Pharmaceutical. Indomethacin-Betamethasone-Methocarbamol under the trade name Ardosons by Chemical Laboratories SON’S ($m_{1}$) and eneric drug by Maver Laboratories ($m_{2}$).

### 2.2. Drop Evaporation

Drug droplets of 2 μL were placed onto glass slides of 22 mm × 22 mm. The cover glasses were manufactured by VELAB (model VE-C22 with thickness of 0.006${}^{\prime \prime }$). They were made of transparent, pure borosilicate glass, resistant to chemicals, free of dust and grease. The cover glasses were washed with distilled water. The volume of the drops was controlled using a micropipette. The droplets were evaporated at substrate temperature $T_{s}$ = 63 ${}^{\circ }$C; and relative humidity (RH) of 25%. The water activity effect was used to control the relative humidity: we placed silica gel (Sigma-Aldrich, St. Louis, MO, USA,13,767) and water in two different containers of different volume to reach the relative humidity (RH) of 25%. The substrate temperature was controlled by a thermoelectric cell with Peltier effect. The high substrate temperature at 63 ${}^{\circ }$C was selected in order to accelerate the evaporation process. It was reported that an increase of 38 ${}^{\circ }$C in the substrate temperature, from 25 to 63 ${}^{\circ }$C, reduces by over 16 times the patterns formation time of deposits (from 1600 s to 100 s) [51].

### 2.3. Image Acquisition

The drug droplets were observed using a microscope (Velab, VE-M4, 4x and 10x). Patterns formation was registered using a digital camera (Nikon Digital, SLR Camera D3200). The image acquisition of dried droplets of drugs was carry out after the complete evaporation. The image resolution was 5470 × 4000 pixels.

### 2.4. Texture Analysis

The texture analysis is based on gray level co-occurrence matrix, in which the number of rows and columns is associated to the number of gray levels ($N_{g}$) [24,52,53]. To calculate a likely variation between gray level *i* and *j* in a displacement distance (*d*) and angle (ϕ) we used the matrix element p(i,j) given by:

(1) $$p\left(i,j\right)=\frac{C(i,j)}{\sum_{i=0}^{N_{g}-1}\sum_{j=0}^{N_{g}-1}C\left(i,j\right)}.$$

C(i,j) represent the number of events of gray levels *i* and *j* in the virtual measurement window of the (d,ϕ) pair. Observe that p(i,j) is delimited by a limit given by the $N_{g}$×$N_{g}$ size.

We estimated the mean and the standard deviation of the columns and rows as follow:

(2) $$u_{x}=\sum_{i=0}^{N_{g}-1}\sum_{j=0}^{N_{g}-1}i\cdot p\left(i,j\right),\;\sigma_{x}=\sum_{i=0}^{N_{g}-1}\sum_{j=0}^{N_{g}-1}{\left(i-u_{x}\right)}^{2}\cdot p\left(i,j\right),$$

(3) $$u_{y}=\sum_{i=0}^{N_{g}-1}\sum_{j=0}^{N_{g}-1}j\cdot p\left(i,j\right),\;\sigma_{y}=\sum_{i=0}^{N_{g}-1}\sum_{j=0}^{N_{g}-1}{\left(j-u_{y}\right)}^{2}\cdot p\left(i,j\right).$$

x,y represents the columns and the rows, respectively. According to these equations, the texture parameters of GLCM are:

#### 2.4.1. Angular Second Moment (ASM)

(4) $$E=ASM=\sum_{i=0}^{N_{g}-1}\sum_{j=0}^{N_{g}-1}p{\left(i,j\right)}^{2}.$$

*E* is the angular second moment (Energy), and measured of global homogeneity of a digital image (texture coarseness). An image is textural uniformity at higher energy values. In contrast, at lower energy, the texture of an image is completely heterogeneous.

#### 2.4.2. Moment of Inertia

(5) $$I=\sum_{i=0}^{N_{g}-1}\sum_{j=0}^{N_{g}-1}{\left(i-j\right)}^{2}p\left(i,j\right).$$

*I* is the Inertia, and compute the roughness in an image i.e., *I* increase with the number of pixels in high contrast.

#### 2.4.3. Correlation

(6) $$COR=U=\frac{\sum_{i=0}^{N_{g}-1}\sum_{j=0}^{N_{g}-1}\left(ij\right)\cdot p\left(i,j\right)-u_{x}u_{y}}{\sigma_{x}\sigma_{y}}.$$

Gray-level of some regions in an image are similar at high correlation values. In contrast, at lower *U* values, gray-level of regions in an image are different.

#### 2.4.4. Inverse Difference Moment (IDM)

(7) $$IDM=\sum_{i=0}^{N_{g}-1}\sum_{j=0}^{N_{g}-1}\frac{1}{1+{\left(i-j\right)}^{2}}p\left(i,j\right).$$

IDM is the inverse difference moment. High IDM values mean high local homogeneity of an image, while low IDM values indicate local inhomogeneity.

#### 2.4.5. Entropy

(8) $$H=-\sum_{i=0}^{N_{g}-1}\sum_{j=0}^{N_{g}-1}p\left(i,j\right)log\left(p\left(i,j\right)\right).$$

*H* is the entropy, and estimates the randomness of the image texture. An image has large heterogeneous regions in an image at high *H* values. In contrast, at low *H* values, an image has small heterogeneous regions. We carried out texture analysis over deposits groups of 16 elements using 8 bit images, at a particular displacement distance *d* = 2 and angle ϕ = 0.

### 2.5. The Receiver Operating Characteristic (ROC) Curve

The Receiver Operating Characteristic (ROC) curve is used to quantify the accuracy of a test to discriminate between elements of two groups [14,54,55,56]. The complete separation between two distributions evidences a perfect diagnosis, while partial overlap implies lower effectivity of the test [57]. By tradition, the ROC curve is obtained by plotting the sensibility (true positive rate) as a function of 1-Specificity (True Positive rate) for each possible cut-off value. Sensitivity is the probability that a test correctly classifies an element as positive. Mathematically, this quantity is writing as follows:

(9) $$Sensitivity=\frac{TP}{TP+FN},$$

where TP and FN are the number of true positives and false negatives, respectively. Specificity (True Negative rate) is the probability of classifying an element as negative. Mathematically, this quantity is defined as:

(10) $$Specificity=\frac{TN}{TN+FP},$$

where TN and FP are the number of true negatives and false positives, respectively. 1- sensibility (False Positive rate) is the probability of classify as false positive an element. Mathematically, this can be expressed as:

(11) $$1-Specificity=\frac{FP}{FP+TN}.$$

Therefore, the ROC Curves are a graphical representation of the relationship between the True Positive rate and the False Positive rate, and they are contained in $\left[0,1\right]$x$\left[0,1\right]$ because both, the ordinate and abscissa axes, are probabilities. Each point on the ROC curve is a cutoff point used to designate test-positive. Finally, the area under the curve ROC (AUC) is the probability that the algorithm classifies correctly a positive element. AUC calculates the accuracy of a diagnostic test, and it is easily computed by the trapezoidal rule [58]. The detailed procedure of ROC curve analysis can be found in [57].

## 3. Results

### 3.1. Pattern Formation in Medicines by Droplet Evaporation

**Figure 1:**
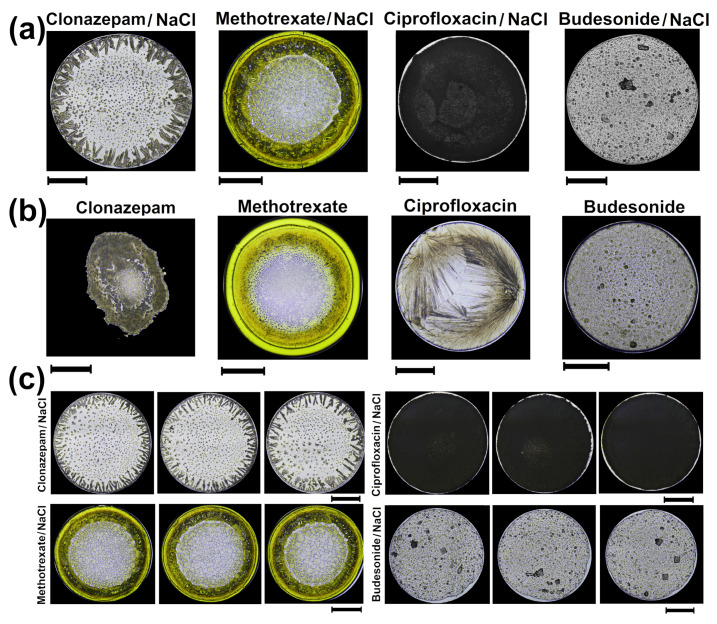
Patterns in dried droplets of medicines (**a**) Patterns of dried droplets of Clonazepam, Methotrexate, Ciprofloxacin and Budesonide with NaCl formed at 63 ${}^{\circ }$C (RH = 25%). (**b**) Patterns of dried droplets of Clonazepam, Methotrexate, Ciprofloxacin, and Budesonide without NaCl formed at 63 ${}^{\circ }$C (RH = 25%). (**c**) The high reproducibility in the pattern formation of Clonazepam, Methotrexate, Ciprofloxacin and Budesonide with NaCl. The length of the black lines indicate 1 mm.

Figure 1a shows patterns of dried droplets of Clonazepam, Methotrexate, Ciprofloxacin, and Budesonide with NaCl (ϕ = 0.5%) formed on a hot substrate at 63 ${}^{\circ }$C. The Clonazepam/NaCl structures exhibit a crown needle-like pattern pointing inwards. It is noted that the aggregates in numerous star-like crystals of different sizes heterogeneously are distributed in the central region of the deposits. Dried droplets of Methotrexate/NaCl show dendritic branched crystallization patterns surrounded by a thin smooth coffee ring. Between the coffee ring and the inner region, a dense film accumulation in a crown shape is observed. In contrast, deposits of Ciprofloxacin/NaCl formed are rough without complex aggregates or a coffee ring. Dried droplets of Budesonide/NaCl form some large and amorphous crystals surrounded by bump-like pattern.

We investigated the pattern formation of dried droplets of medicines without NaCl in order to explore the role of NaCl in the formation of complex aggregates. Figure 1b shows patterns of dried droplets of Clonazepam, Methotrexate, Ciprofloxacin, and Budesonide formed on a hot substrate at 63 ${}^{\circ }$C. There are clear differences between groups of deposits with salt and without salt. Interestingly, Clonazepam droplets cannot be pinned, forming an amorphous puddle over the substrate. It is observed that the terminal edge of the stain is distorted and that its entire area exhibits an irregular rough-texture with small rough bump-like patterns. Therefore, the radial symmetry of the deposits can increase when NaCl is added to the drug drops placed on a substrate at $T_{s}$ = 63 ${}^{\circ }$C. It occurs because the ionic concentration gradients promote the formation of a coffee ring that serves as an anchor to fix the contact line of the droplet. On the other hand, Methotrexate droplets without salt form a smooth coffee ring. A coarse film accumulation separation, in a crown shape, is observed between the coffee ring and the inner structure. The center region of the drop shows a bump-like pattern. There are no significant changes in the salt-free patterns formed by Budesonide. Finally, the high reproducibility of dried drops of drugs with NaCl is depicted in Figure 1c.

### 3.2. Texture Analysis and Classification of Dry Droplet Patterns

**Figure 2:**
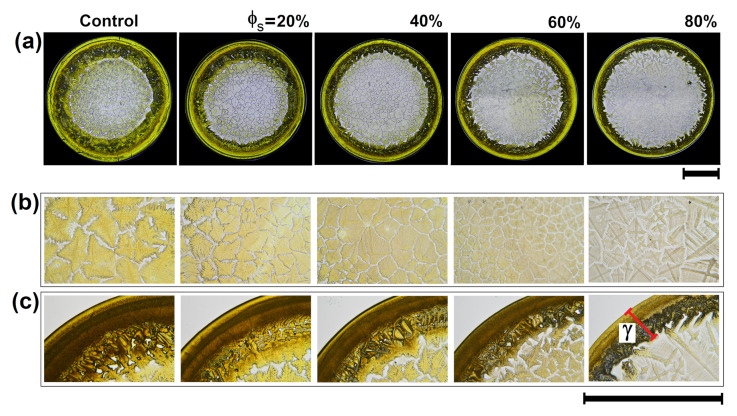
Deposits obtained from diluted Methotrexate with tap water. (**a**) Methotrexate/NaCl deposits produced by mixing drug solutions with varying ratios of tap water. Droplet evaporation was carried out on a solid surface at $T_{s}$ = 63 ${}^{\circ }$C and RH = 25%. (**b**) Zoom at central region of the deposits. (**c**) Zoom at representative crown region of the deposits. The length of the black lines indicate 1mm. The red line shows the crown thickness γ. For each pattern, γ is the average of 8 measurements in different regions in the crown.

Figure 2 shows the patterns formed by the evaporation of drops of diluted Methotrexate with tap water. A naked-eye observation indicates that all patterns of dried droplets preserve similar structural characteristics, irrespective of the dilution level. However, a closer inspection reveals two important structural changes on the stains: the morphology of the crystals in the center changes (Figure 2b) and the crown thickness (γ) decreases (Figure 2c). At higher level of water dilution, the morphology of the crystals changes from dense to simple dendritic branched crystallization. This occurs simply because water changes the mass transport mechanisms that affect the final aggregation processes on the substrate, specially, in the crown region.

Structural analysis based on the gray level co-occurrence matrix (GLCM) quantifies the texture of dried deposits. We must remark, however, that this texture analysis can be carried out on different regions of the deposit. The choice of regions to be analyzed depends on the morphological characteristics of each set of drug stains. For example, in the case of Methotrexate, we can carry out texture analysis on the crown region.

**Figure 3:**
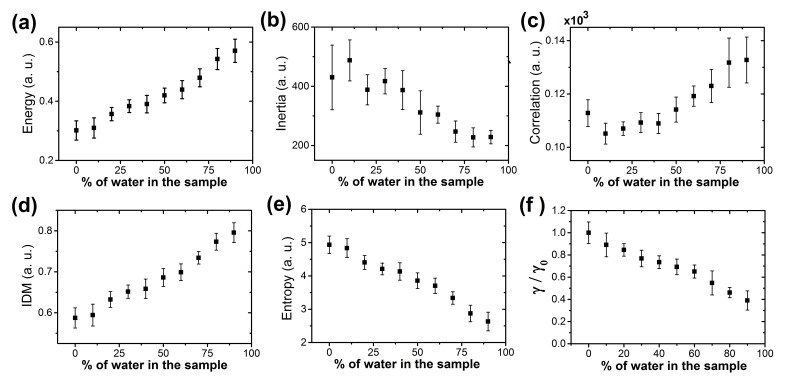
Texture analysis of dried droplets of Methotrexate/NaCl. The GLCM parameters ((**a**) Energy, (**b**) Inertia, (**c**) Correlation, (**d**) IDM and (**e**) Entropy) of Methotrexate/NaCl patterns produced with varying ratios of tap water at $T_{s}$ = 63 ${}^{\circ }$C and RH = 25%. (**f**) The normalized crown thickness $\gamma /\gamma_{0}$ of patterns in dried droplets of Methotrexate/NaCl as a function of ratios of tap water at $T_{s}$ = 63 ${}^{\circ }$C and RH = 25%. Here, $\gamma_{0}$ is the crown thickness of Methotrexate/NaCl pattern formed without the addition of any agent. The error bars are standard deviations for n= 16.

Figure 3a–e shows the GLCM values for the crown region of Methotrexate deposits generated at different concentrations of tap water. The energy, which captures the global homogeneity of deposits, changes from heterogeneity to textural uniformity, see Figure 3a. The roughness and complexity of texture deposits, estimated by the Inertia, decrease with percentage of water dilution, see Figure 3b. Figure 3c shows that the Correlation, which measure the degree of similarity between GLCM elements, evolves from different to similar gray-level regions. Moreover, the characteristics of inhomogeneous deposits changes to local homogeneity, see IDM in Figure 3d. Finally, the Entropy, which captures the heterogeneous regions in the deposits, decrease with percentage of dilution level see Figure 3e. Clearly, these texture changes occur because the concentration of Methotrexate decreases as the dilution with tap water increases.

Moreover, it is interesting to note that at first glance, the crown thickness γ can be used to identify Methotrexate deposits containing 20% tap water and higher, as shown in Figure 3f. In addition to the reduction of γ, correlated to the drug concentration, this result means that simple structural parameters may potentially be used as an effective quality control marker.

**Figure 4:**
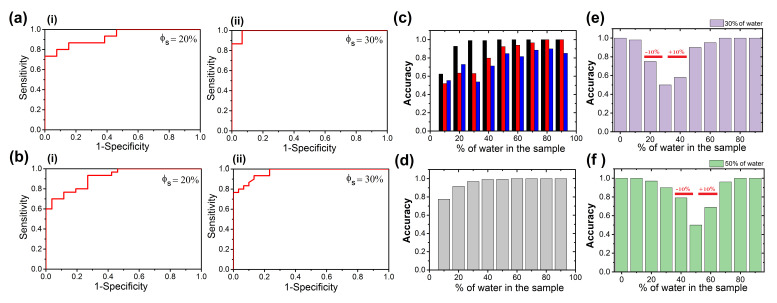
Receiver Operating Characteristic (ROC) curves. (**a**) (ROC) curves plotted from the entropy analysis of the crown in Methotrexate/NaCl patterns formed with varying ratios of tap water (**i**) 20, and (**ii**) 30%, respectively. (**b**) The ROC curves plotted from analysis of the normalized crown thickness $\gamma /\gamma_{0}$ of Methotrexate/NaCl patterns produced with varying ratios of tap water (**i**) 20, and (**ii**) 30%, respectively. (**c**) Accuracy for entropy estimated in different regions of Methotrexate/NaCl patterns: the crown (black), the center (red), and the complete deposit (blue). (**d**) Accuracy for the normalized crown thickness $\gamma /\gamma_{0}$ of Methotrexate/NaCl patterns. Accuracy for classifying groups of drug diluted with water at (**e**) 30% and (**f**) 50% by using entropy analysis of the crown in Methotrexate/NaCl patterns.

In order to estimate the effectiveness of the droplet evaporation method as a quality control marker of medicines, we used the receiver operating characteristic (ROC) curves. Figure 4a shows two ROC curves calculated from the entropy values of the crown stain for 20 and 30% of tap water. With this test, we determine the optimal sensitivities and specificities from the ROC data. First, we determine the sum of sensitivity and specificity; thereafter, we consider specificity values larger than 50%. Finally, we extracted the value with the highest sensitivity. The optimal sensitivities and specificities from these ROC data are 0.94, 0.83; and 0.88, 0.66, respectively. Two ROC curves related to the crown thickness γ of patterns in dried droplets of Methotrexate with 20 and 30% of water dilution are shown in Figure 4b. For these cases, the optimal sensitivities and specificities are 1, 0.73; 1, 0.93; 0.7, 0.96; and 0.8, 0.96, respectively.

The area under the ROC curve (AUC) can be used to quantify the discriminative capacity of our diagnostic test. This quantity provides the probability that an element of the set of dried droplets produced by diluted medicines can be correctly classified. Figure 4c shows the accuracy of Entropy calculated in three different regions in patterns of dried droplets of Methotrexate: the interior of the droplet (blue), the crown (red), and the complete deposit (black). The entropy associated with the crown and the center of the patterns of dried droplets of Methotrexate increases with concentration, while the entropy calculated from the entire deposit does not have a particular trend. The lack of a discernible trend in the entropy of the entire deposit results from the different morphological changes in the different regions of the droplet. For example, thickness of the crown and ring decreases, while the size of the crystals increases, and they evolve from dense to simple dendritic branched patterns (See Figure 2). The greater accuracy was found in Entropy values estimated in the crown region (values above 0.95, with 20% of water). The entropy values estimated by the complete deposit gives the lower AUC values, (close to 0.6, with 20% of water). Figure 4d shows the accuracy of the crown thickness γ of patterns in dried droplets of Methotrexate. The γ values give high AUC values, from 0.77 to 0.95, with 10 and 20% of water, respectively. Table 1 depicts, for the three deposit regions, the AUC values for the different texture parameters.

In order to explore the capability of the method to identify the dilution percentage of a drug, in Figure 4e,f we depicted accuracy for entropy (captured in the crown) to classify drugs diluted with water at 30% and 50%. The method loss discriminatory capacity to identify two groups of diluted drugs in a range of percentage of water from −10% to +10%. Here, the accuracy is lower than 0.75. In contrast, outside these limits, the accuracy is above 0.9.

**Figure 5:**
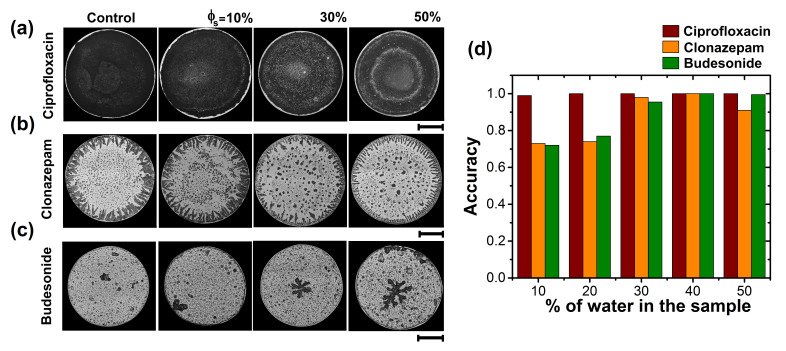
Recognition of Criprofloxacin, Clonazepam, and Budesonide. Patterns of dried droplets of (**a**) Criprofloxacin/NaCl, (**b**) Clonazepam/NaCl, and (**c**) Budesonide/NaCl produced by different dilutions of tap water. Patterns formation was carried out at $T_{s}$ = 63 ${}^{\circ }$C, and RH = 25%. The length of the black lines indicate 1 mm. (**d**) Accuracy for different medicines: Criprofloxacin/NaCl (red), Clonazepam/NaCl (orange), and Budesonide/NaCl (green). Texture analysis was carried out using different parameters: entropy of the entire deposit, entropy at the center of the deposit, and the superficial area of the aggregate formed in the center of the deposit, respectively.

**Figure 6:**
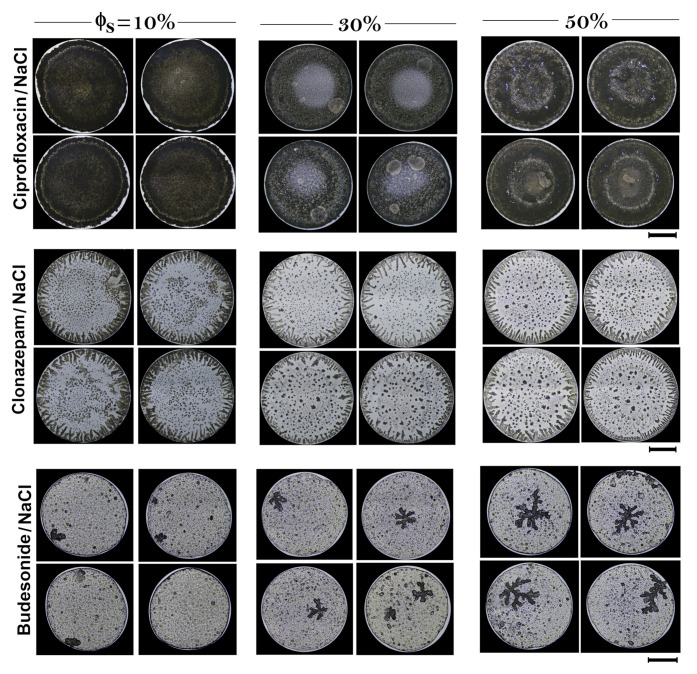
Reproducibility of patterns in dried droplets of Criprofloxacin/NaCl, Clonazepam/NaCl, and Budesonide/NaCl produced by different dilutions of tap water. Patterns formation was carried out at $T_{s}$ = 63 ${}^{\circ }$C, and RH = 25%. The length of the black lines indicate 1 mm.

To assess the potential use texture analysis of dried droplets as a quality control method for different drugs, we analyzed Ciprofloxacin, Clonazepam, and Budesonide, which are medicines associated with different routes of administration. Figure 5a–c shows the patterns formed during drying of drops of diluted drugs with tap water. Different parameters were used to analyze the structural characteristics of each dried droplet: (1) entropy of the entire deposit; (2) entropy in the center of the deposit; and (3) the superficial area of the aggregate formed in the center of the deposit, respectively. Figure 5d. shows the accuracy of texture analysis in dried deposits. Depending on the drug, texture analysis of stains is capable to identify dilution with 10% of water, with an accuracy from 72 to 99%. Patterns of dried droplets of Ciprofloxacin are highly dense uniform deposits. The addition of water allows the formation of small, low-density regions that coexist within the high-density deposit. This high contrast texture of the Ciprofloxacin surface facilitates the efficient detection of Ciprofloxacin dilution with tap water (accuracy above 99%), even at low concentrations (10%). Note that the method distinguishes dilution in medicines with 30% of tap water, with an accuracy greater than 95%. The reproducibility of dried drops of drugs diluted with tap water is depicted in Figure 6.

**Figure 7:**
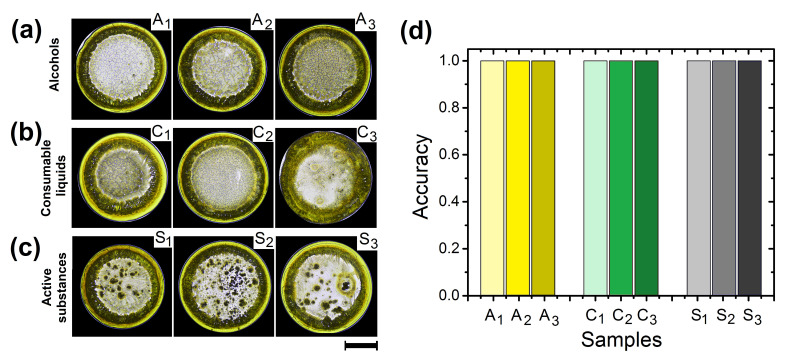
Recognition of Methotrexate containing different substances Patterns in dried droplets of Methotrexate/NaCl produced by the addition of 10% of (**a**) alcohols (ethanol($A_{1}$), methanol($A_{2}$), and isopropanol($A_{3}$)), (**b**) consumable liquids (rehydrating serum($C_{1}$), carbonated drink($C_{2}$), and liquid detergent ($C_{3}$)), and (**c**) medicines (Folic acid($S_{1}$), Diclofenac ($S_{2}$), and Acetaminophen($S_{3}$)). The relative concentration of the three medicines and Methotrexate in the final sample is 1:10, *w*/*w*. The control parameters in pattern formation from the evaporation of droplets were: $T_{s}$ = 63 ${}^{\circ }$C, and RH = 25%. The black line indicates 1 mm. (**d**) The corresponding Accuracy for the three groups of substances based on the entropy of the entire deposits.

We also conducted experiments with consumable liquids and active substances to explore the capability of the method as a tool for the quality control of medicines. Patterns in dried droplets of Methotrexate produced with 10% ethanol, methanol, and isopropanol show similar crowns but different structures in the inner region, see Figure 7a. At the center of dried deposits, ethanol produces aggregates with few crystals, while isopropanol promotes the formation of aggregates with many crystals. In the context of pattern formation in dried droplet, alcohols act as a surfactant that create surface gradients changing the inner flows in the droplet [59,60]. This, in turn, modifies the concentration gradients to produce amorphous aggregates at the center of the droplet. Figure 7b shows patterns of dried droplets of Methotrexate produced with 10% rehydrating serum, carbonated drink, and liquid detergent. The more evident structural changes in dried deposits of Methotrexate are as follow: rehydrating serum generate large crystals surrounded by a thick coffee ring, carbonated drink produce small interlocked crystals aggregates at the center of dried deposits, and liquid detergent does not form a coffee ring stain and small crystals at the center of the deposits. This is because detergents can abruptly change mass transport mechanisms and colloids modify the aggregation process among components of the droplet [61].

Figure 7c shows patterns in dried droplets of Methotrexate produced with 10% of Folic acid, Diclofenac and Acetaminophen. The formation of amorphous aggregates is the most evident change generated by these medicines in dried deposits of Methotrexate. Methotrexate is an antineoplastic drug and an antimetabolite of Folic acid, both substances are structural analogues with similar molecular weights [62,63]. Because of this, Methotrexate is a competitive inhibitor of many enzymes that use folates (the different forms of Folic acid). Diclofenac and Acetaminophen are non-steroidal anti-inflammatory drugs (NSAIDs) with analgesic effects [64,65,66]. NSAIDs drugs are amphiphilic molecules, this means that they contain hydrophilic and hydrophobic groups. Interestingly, Methotrexate and Folic acid are molecules that can be considered as amphiphilic when their glutamic acid residue is deprotonated (at higher pH) [67]. Moreover, the hydrogen bond acceptor sites of Diclofenac and Acetaminophen are low compared to Methotrexate molecule.

The macromolecular interactions between drugs in an aqueous medium involve complex processes and a large number of biochemical mechanisms [68]. In the context of drop evaporation, the interaction between Methotrexate and the other medicines might be regulated by the H bond acceptor sites and hydrophobic/hydrophilic characteristic of the active pharmaceutical ingredient. The amphiphilic nature of Acetaminophen and Diclofenac could reinforce their hydrophobic interaction as the water evaporates. This can lead to their aggregation on the droplets and provide a relative freedom to Methotrexate-water interaction.

Since water molecules have amphoteric characteristics, they are more likely to interact with the 12 sites of H-bonding of Methotrexate than the sites of Diclofenac and Acetaminophen, 3 and 2 respectively. On the other hand, although the H-bond acceptor sites of Folic acid is 10, the concentration of Methotrexate in the sample that we used is ten times bigger. Therefore, most of the interactions are mediated by Methotrexate-water molecules and not by interactions that include Diclofenac, Acetaminophen, and Folic acid. As result, these medicines aggregate to form small clusters at the center of the droplet. Figure 7d. shows the accuracy of Entropy in dried deposits of Methotrexate. Patterns of Methotrexate with 10% of alcohols, consumable liquids, and therapeutic products can be differentiated from Methotrexate deposits with an accuracy greater than 99%. Note that, using the same protocol, dried deposits generated with 10% tap water are differentiated with an accuracy of less than 60%.

**Figure 8:**
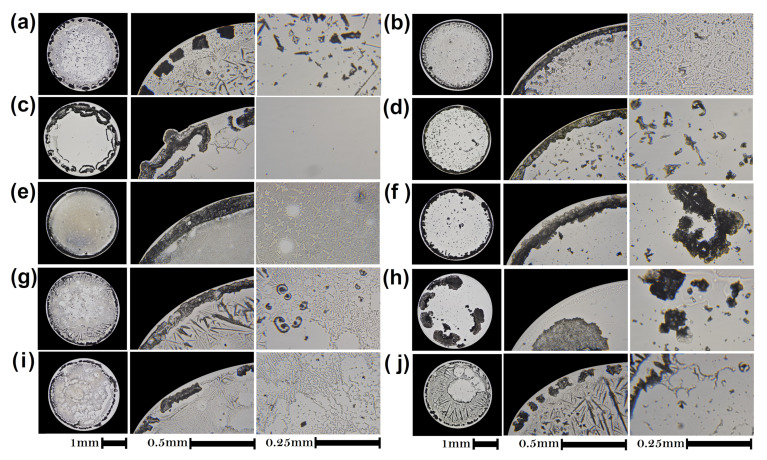
Patterns in dried droplets of 10 medicines. Dried droplets of (**a**) Pantoprazole/NaCl, (**b**) Fluconazole/NaCl (**c**) Clindamycin/NaCl, (**d**) Nitrofurantoin/NaCl, (**e**) Cefaclor/NaCl, (**f**) Metronidazole-Diiodohydroxyquinoline/NaCl, (**g**) Meloxicam-Methocarbamol/NaCl, and (**h**) Naproxen-Carisoprodol/NaCl. Deposits of Indomethacin-betamethasone-methocarbamol/NaCl of two different manufacturers (**i**) $m_{1}$ and (**j**) $m_{2}$, respectively.

To prove that different drugs generate different patterns, Figure 8 shows dried droplets of ten medicines. All patterns in dried droplets of drugs are different. Pantoprazole/NaCl deposits contain a coffee ring formed of small aggregates, see Figure 8a. Inside the deposit, we observed small smooth regions enclosed by an arborescent crystalline pattern that resembles a fern frond. Figure 8b shows that the coffee ring of Fluconazole/NaCl deposits is composed of small groups of aggregates. In the central region of the dried droplet, there are large smooth regions surrounded by needle-shaped crystals of varying size. Figure 8c shows Clindamycin/NaCl deposits formed by a uniform coating with large amorphous aggregates located on the periphery. In contrast, Figure 8d shows that Nitrofurantoin/NaCl dried droplets exhibit a coffee ring, while the inner region of the deposit shows small formations of amorphous crystals. Cefaclor/NaCl droplets form deposits with a well-defined coffee ring, see Figure 8e. In the internal part of the deposit are small aggregates that surround the smooth regions. Figure 8f shows that Metronidazole–Diiodohydroxyquinoline/NaCl deposits are formed by an amorphous coffee ring and crystalline aggregates of different sizes that cover the entire internal region of the deposit. Meloxicam-Methocarbamol/NaCl deposit shows a well-defined coffee ring and needle-like aggregates, see Figure 8g. The interior of the deposit is made up of smooth regions, aggregates in the form of dendrites and amorphous aggregates. Figure 8h shows that Naproxen-Carisoprodol/NaCl deposits exhibit large amorphous aggregates located in different areas of the deposit.

With the aim of knowing wheher two medicines, with the same active ingredient, produce the same characteristic patterns, in Figure 8i,j we depicted deposits of Indomethacin-betamethasone-methocarbamol/NaCl of two different manufacturers. Although these patterns show similarities, they are different. Figure 8i shows a deposit with a coffee ring made up of a discontinuous line of various aggregates located on the contact line. Inside the deposit it is possible to observe small flat regions, amorphous aggregates and star-shaped aggregates. In contrast, Figure 8j shows a deposit with a well-defined coffee ring. The internal region of the deposit is totally covered by small aggregates. Since both medicines contain the same active ingredient, the differences found in both dry drops may be due to the vehicle.

### 3.3. Patterns Formation of Dried Droplets of Medicines: Physical Mechanisms

There is no general model capable to predict the flow within the droplet and aggregation mechanisms that give rise to pattern formation from drying. One of the greatest difficulties in predicting pattern formation is that the bio-molecules inside a drop can act as surfactants. These molecules influence the surface tension on the contact surface between two phases [69] and can dramatically modify mass transport via Marangoni flows [70]. These flows can emerge through convection flow, Marangoni-Benard convection (where periodic hexagonal network forms) [71,72], and Marangoni eddies [48]. Therefore, the growth process of patterns can be different for each surfactant [73,74,75]. Depending on the surfactant type (anionic or cationic) and the type of particles, the edge depositions can be suppressed [47] or homogeneous patterns can be induced in a certain range of concentration [47,76].

In addition to the role that surfactants play in flow mechanisms, another difficulty in predicting pattern formation is the collective phenomena that arise from the coexistence of molecules of different species. For example, consider the evaporation of a droplet of lysozyme and bovine serum albumin [77]. In this case, the lysozyme molecules arrive first to the center of the substrate deposit on the substrate due to their low-molecular weight; however, they are displaced by relatively larger proteins of BSA, which arrive later. In this case, the lysozyme molecules arrive first to the center of the droplet to form a film due to their low-molecular weight; however, they are displaced by larger proteins of BSA, which turn up later. This effect, the so-called Vroman effect, avoids crack formation in the central region of the film [26,77]. Moreover, if the lysozyme intervenes during the aggregation of BSA, it may be trapped within the interactions of subunits of hydrophobic surfaces, culminating in the formation of dendritic structures, undulated branches, and interlocked chains [26].

In addition to the problems mentioned above, the prediction of pattern formation in medicines poses additional challenges. For drugs, medicinal or life-style, the active ingredient most of the time is accompanied by a vehicle, which is an inert liquid substance that is used in pharmaceutical formulations to reach a certain volume or density. The vehicle may or may not be accompanied by other excipients, which are substances that modify some of the characteristics of the active ingredient in the final product. In general, the exact formulation of both the vehicle and the excipients is not of public knowledge. Without knowing these composition details, it is not possible to even attempt prediction of the formation of dry droplet patterns.

**Figure 9:**
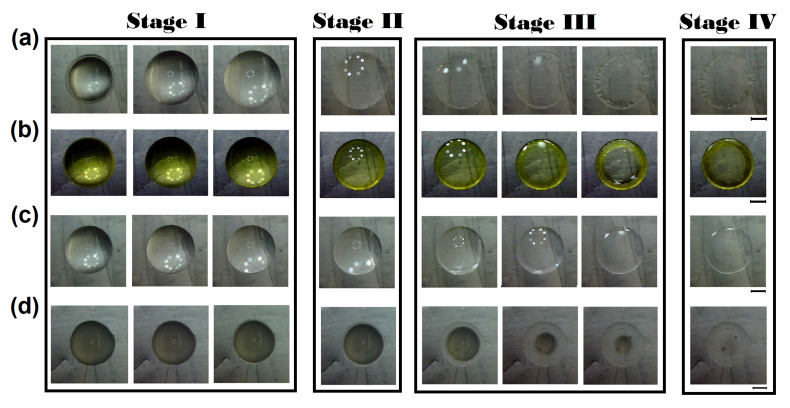
Pattern formation of drug deposits. Pattern formation of (**a**) Clonazepam/NaCl (t = 0, 45, 300, 1650, 1800, 1950, 2100, and 2130 s), (**b**) Methotrexate/NaCl (t = 0, 30, 60, 135, 150, 165, 180, and 240 s), (**c**) Criprofloxacin/NaCl (t = 0, 30, 45, 60, 75, 90, 105, and 150 s), and (**d**) Budesonide/NaCl (t = 0, 30, 45, 60, 105, 165, 180, and 240 s). Labels I, II, and III indicate three stages of formation: increasing in contact area of the droplet, recirculating flow, and the emergence of the stain pattern, respectively. Label IV corresponds to the residual pattern deposit. The diameter of the final deposits are 3.6, 3.15, 3.25, and 3.2 mm, respectively. Pattern formation from the evaporation of droplets was carried out at $T_{s}$ = 63 ${}^{\circ }$C, and RH = 25%.

Despite all the difficulties, we can clearly identify that pattern formation from the evaporation of drug drops occurs in three main stages. A sequence of images of such a process is shown in Figure 9. In the first stage of evaporation, the contact line of the droplet expands outwards, broadening the contact area between the droplet and the surface, see Stage I in Figure 9. A first inspection indicates that Clonazepam drops extend more than any of the other medicines considered. The differences in extension also indicate different values of surface properties for these medicines, which most likely result from the different compositions. In the second stage of evaporation, capillary and Marangoni flows compete to establish the flow inside the drop, as shown in Stage II, Figure 9. Without measurements of the flow inside the drop, which would be technically difficult, it is unfathomable to discern the details of this process, but some aspects can be inferred from the subsequent evaporation phase. In the third stage of evaporation, in droplets containing Clonazepam, Methotrexate, and Ciprofloxacin, the capillary flows overcome Marangoni flows, see Stage III in Figure 9. Here, evaporation at the edge of the droplet induces outwards flow that increases the concentration of compounds at the rim of the drop (the coffee ring) ring [42,43,44].

It is interesting to note that, for Ciprofloxacin, the coffee ring is not observed. Sodium chloride has a specific interaction with Ciprofloxacin. The NaCl-Cipro interaction has been reported to lead to the formation of small crystalline cubes suggesting that sodium chloride ions encapsulate the active ingredient [78,79]. This process may inhibit the hydrophobic interactions of Ciprofloxacin causing a change in the interaction of the various elements that contain the excipient of this therapeutic product. As a result of this complex interaction, Marangoni flows can emerge to avoid the coffee ring effect. Interestingly, for droplets of Budesonide, the fluid (in a dome form) moves toward the inner part of the drop, see Figure 9d. This mass transport mechanism commonly appears after the protein gelation process [26]. Finally, the residual structure of drugs and salt emerges during Stage IV, as shown Figure 9.

In NaCl solutions, water dissolves the ionic compound due to the electrical charges of Na${}^{+}$ and Cl${}^{-}$. The negatively-charge side of water molecules surround the sodium ions, and the positively-charge side of water molecules surround the chloride ions. During the evaporation of a droplet, ions are transported not only by the capillary force, but also by Marangoni flows. As the drop evaporates, the solute concentration increases in time. The formation of complex aggregates of NaCl is related to surface nucleation and aggregation processes. The creation of a cluster of critical size occurs at imperfections on a surface when the bulk contribution surpasses that of the surface, and then the nucleus becomes stable. The rapid growth of NaCl crystals is triggered once a saline solution overcomes the limit of solute (NaCl) that the solvent (water) can admit. The salt crystals formed on a surface consist of a face-centered cubic (fcc) lattice of Na${}^{+}$ ions and another fcc of Cl${}^{-}$ ions, together forming a simple cubic lattice with alternating Na${}^{+}$ and Cl${}^{-}$ ions. In general, regardless the NaCl solution, high NaCl concentration induces larger crystals while small concentrations produce small aggregates. However, the final morphological characteristics of the pattern in dried droplets depend on the interaction among Na${}^{+}$ and Cl${}^{-}$ ions and the components of a solution [80]. Under this scenario, the crystals growing from complex aggregates depend on the surface type of bio-film [81], and the concentration gradients which determine the degree of supersaturation across the droplet. There are many examples of this process: protein films [81], pattern of dried droplets in alcoholic drinks [39], food deposits [17], and spermatozoa stains [14].

## 4. Discussion

We used Clonazepam/NaCl, Methotrexate/NaCl, Ciprofloxacin/NaCl, and Budesonide/NaCl to quantify the performance of texture analysis of dried droplets for the quality control of medicines with different routes of administration. Clonazepam is administered orally, and it is used to prevent and treat seizures, involuntary muscle spasms, panic disorder, and anxiety disorders. Methotrexate can be administered parenterally, and it is an important antineoplastic used to treat breast and lung cancers, some types of lymphoma, and leukemia. Ciprofloxacin can be a topically administered ocular drug. This medicine is a broad spectrum antibiotic that is used to treat or prevent bacterial infections such as conjunctivitis, pneumonia, urinary tract infection among other diseases. Finally, Budesonide can be administered nasally, and it is a glucocorticoid used to treat allergic rhinitis and nasal polyposis.

The entropy is shown to be the best texture parameter for differentiation among deposits. This occurs because the number of heterogeneous regions in an image is mostly affected by the formation of salt aggregates and inhibition of crowns. We must remark, however, that image analysis based on GLCM differs from the analysed region in the deposit. For example, for Methotrexate, texture analysis applied to the entire deposit provides lower yields compared to the crown region. Moreover, we found that differentiation between patterns can be greatly improved when the classification by groups is based on particular structural characteristics of each deposit. The crown thickness (γ) and the superficial area of the central dried deposit, for Methotrexate and Budesonide, respectively, are examples of that (see Table 2). This means that to increase the efficiency in the quality control of medicines, each deposit must be analyzed considering specific structural aspects. This can only be achieved by a case-by-case examination. However, this process is not new in the context of the quality control of consumable products. Fourier transform infrared (FTIR) spectroscopy and Raman spectroscopy are examples such practices [82,83].

The effects of Raman scattering and IR light, irradiated in a sample, are highly sensitive to detecting the different vibrational modes of the molecules that make up a sample. This is because the symmetry and chemical bonds of a molecule have a specific vibratory frequency. In this way, individual bonds, bond groups, rings, and polymer chains can be identified by a spectrum composed by series of peaks or curves that show the intensity and wavelength of scattered light. Unfortunately, in the analysis of a consumable product, not all regions of the spectrum are useful for identifying impurities and dilutions. For example, water masks molecules present in an aqueous solution over a wide wavelength range of the infrared [84]. Therefore, to carry out a quality control analysis using these analytical techniques, the wavelength range where the spectrum can be associated to the consumable product of interest must be known a priori [85,86,87]. The method of texture analysis of patterns in dried droplets as a method to quality control in medicines is based on an analogous idea. Each medicine must be associated, a priori, with a pattern of dried droplets, instead of a spectrum. Under this scheme, impurities and dilutions promote morphological changes in the patterns, such as suppression or formation of aggregates.

There are some important points to consider before using the droplet evaporation method to the quality control of medicines. First, the evaporation time must be reduced (from hours to seconds) by enhancing heat transfer, placing the droplet on a hot surface. Second, pattern deposits with radial symmetry must be induced by adding NaCl to the solution. Third, the exact range of drop volumes capable of generating statistically similar patterns is unknown. Fourth, since many drugs preserve the active substance without degradation for years, texture analysis could give low accuracy in the diagnostic test to detect expired medicines. Fifth, the texture of each medicine deposit should be analyzed based on its specific morphological characteristics. For example, the analysis of the crown for Methotrexate provides excellent results to differentiate between groups. In contrast, Budesonide deposits do not contain a crown. Instead, they have a central aggregate that serves as an excellent marker.

Some advantages to the quality control of medicines by the droplet evaporation method can be readily identified. (1) The set-up is simple, sophisticated instrumentation is not needed. (2) Identification tests can be easily implemented in basic laboratories where an optical microscope and a magnetic stirrer with hot plate are available. (3) The implementation of the method does not require highly trained personnel. (4) Texture analysis is not computationally expensive, it can be conducted on an ordinary desktop computer. (5) The method can be used for compounds administered by different routes of medication; and finally, (6) The presence of different components can be identified. Two important limitations of the present method must be mentioned: (1) the method can detect changes by water dilution in any drug, with an accuracy above 95%, only when the addition of tap water is equal (or higher) to 30%; and (2) the method works by contrasting the stain with that obtained from an unaltered drop: drop control sample is needed.

The results presented here show that the droplet evaporation method can be an effective tool for the quality control of drugs. The patterns of dried droplets depend on many parameters such as substrate type, humidity, viscosity, initial volume of the droplet, substrate temperature, among many others. Droplets of the same solution can generate a high diversity of patterns under different values of such parameters. However, if we initially fixed all the parameters, droplets of a particular liquid produce patterns statistically similar to each other. Under this scenario, an unknown agent inside the drop can greatly modify the parameters and cause structural changes in the patterns. We take advantage of this phenomenon to propose a new method for the quality control of drugs. The idea of revealing how patterns of dried droplets of medicines are affected by the different control parameters is very attractive. We plan to conduct such measurements in the future and report the results elsewhere. results elsewhere.

## 5. Conclusions

In conclusion, we have presented a novel methodology, based on a texture analysis of patterns in dried droplets, to the quality control of drugs. Four types of medicine were analysed: Methotrexate, Ciprofloxacin, Clonazepam and Budesonide. Heat transfer and ionic interactions are used to induce well-defined and reproducible patterns. Depending on the drug, patterns in dried droplets show a great diversity of complex aggregates. Examples are many: circular to oval splatters, fern-like islands, crown shapes, crown needle-like and bump-like patterns as well as dendritic branched and star-like crystals. Some structural characteristics, such as the stain diameter and superficial area, as well as the gray level co-occurrence matrix (GLCM), were used to characterize deposits. The texture analysis of patterns in dried droplets detects 10% external agents with an accuracy up to 99%. Finally, as long as we have the pattern in a dried droplet left by a control sample, the method shows a potential use for the quality control of medicines.

## Figures and Tables

**Table 1:** Receiver Operated Characteristic (ROC) curves based on texture parameters estimated on different regions of Methotrexate/NaCl stains. Ps = water content (%). Error = standard error. Asym. prob.: asymptotic probability. LCL: the lower confidence limit. UCL: the upper confidence limit.

CROWN										
	$\gamma /\gamma_{0}$					Energy				
P.S.($\phi_{s}$) %	AUC	Std. Error	Asymptotic Prob	95% LCL	95% UCL	AUC	Std. Error	Asymptotic Prob	95% LCL	95% UCL
10	0.77556	0.05941	2.46× 10${}^{-4}$	0.65912	0.89199	0.60444	0.10853	0.32969	0.39174	0.81715
20	0.91667	0.03496	9.33× 10${}^{-8}$	0.84815	0.98518	0.91282	0.05388	2.09× 10${}^{-4}$	0.80723	1.01841
30	0.96944	0.01734	4.20× 10${}^{-10}$	0.93547	1.00342	0.97778	0.02099	8.24× 10${}^{-6}$	0.93663	1.01892
40	0.99111	0.00874	6.37× 10${}^{-11}$	0.97399	1.00823	0.97333	0.02408	9.99× 10${}^{-6}$	0.92613	1.02053
50	0.99333	0.00647	5.23× 10${}^{-11}$	0.98065	1.00602	1	0	4.59× 10${}^{-6}$	1	1
60	1	0	2.87× 10${}^{-11}$	1	1	1	0	3.07× 10${}^{-6}$	1	1
70	1	0	6.34× 10${}^{-11}$	1	1	1	0	4.59× 10${}^{-6}$	1	1
80	1	0	3.69× 10${}^{-10}$	1	1	1	0	1.13× 10${}^{-5}$	1	1
90	1	0	2.87× 10${}^{-11}$	1	1	1	0	4.59× 10${}^{-6}$	1	1
	Inertia					Correlation				
P.S.($\phi_{s}$) %	AUC	Std. Error	Asymptotic Prob	95% LCL	95% UCL	AUC	Std. Error	Asymptotic Prob	95% LCL	95% UCL
10	0.65778	0.10158	0.14089	0.45868	0.85688	0.87556	0.06812	4.57× 10${}^{-4}$	0.74203	1.00908
20	0.64103	0.11708	0.20523	0.41156	0.87049	0.85641	0.08099	0.00137	0.69767	1.01515
30	0.52889	0.1193	0.78746	0.29507	0.7627	0.73111	0.09476	0.03102	0.54539	0.91683
40	0.62222	0.1084	0.25402	0.40977	0.83468	0.74	0.09276	0.0251	0.55819	0.92181
50	0.79048	0.08772	0.00776	0.61855	0.9624	0.52143	0.11191	0.8443	0.30209	0.74077
60	0.84889	0.08019	0.00113	0.69172	1.00606	0.87556	0.06508	4.57× 10${}^{-4}$	0.748	1.00311
70	0.94286	0.05003	4.93× 10${}^{-5}$	0.84479	1.04092	0.91429	0.0546	1.46× 10${}^{-4}$	0.80727	1.0213
80	0.96111	0.03916	5.12× 10${}^{-5}$	0.88436	1.03787	0.96111	0.03686	5.12× 10${}^{-5}$	0.88887	1.03336
90	0.95238	0.04887	3.38× 10${}^{-5}$	0.85659	1.04817	1	0	4.59× 10${}^{-6}$	1	1
	IDM					Entropy				
P.S.($\phi_{s}$) %	AUC	Std. Error	Asymptotic Prob	95% LCL	95% UCL	AUC	Std. Error	Asymptotic Prob	95% LCL	95% UCL
10	0.61111	0.1088	0.29976	0.39786	0.82436	0.62444	0.1058	0.24549	0.41708	0.83181
20	0.91282	0.054	2.09× 10${}^{-4}$	0.80699	1.01865	0.92821	0.0482	1.20× 10${}^{-4}$	0.83374	1.02267
30	0.97556	0.02247	9.07× 10${}^{-6}$	0.93152	1.01959	0.99111	0.01183	4.58× 10${}^{-6}$	0.96793	1.0143
40	0.97556	0.02247	9.07× 10${}^{-6}$	0.93152	1.01959	0.99111	0.01183	4.58× 10${}^{-6}$	0.96793	1.0143
50	1	0	4.59× 10${}^{-6}$	1	1	1	0	4.59× 10${}^{-6}$	1	1
60	1	0	3.07× 10${}^{-6}$	1	1	1	0	3.07× 10${}^{-6}$	1	1
70	1	0	4.59× 10^−6^	1	1	1	0	4.59× 10^−6^	1	1
80	1	0	1.13× 10${}^{-5}$	1	1	1	0	1.13× 10${}^{-5}$	1	1
90	1	0	4.59× 10${}^{-6}$	1	1	1	0	4.59× 10${}^{-6}$	1	1
CENTER										
	Area					Energy				
P.S.($\phi_{s}$) %	AUC	Std. Error	Asymptotic Prob	95% LCL	95% UCL	AUC	Std. Error	Asymptotic Prob	95% LCL	95% UCL
10	0.89778	0.06096	2.05× 10${}^{-4}$	0.77829	1.01726	0.50222	0.10877	0.98345	0.28903	0.71541
20	0.54872	0.11511	0.66166	0.3231	0.77434	0.62051	0.11223	0.27901	0.40054	0.84049
30	0.77333	0.08836	0.01074	0.60016	0.94651	0.76444	0.0883	0.01359	0.59139	0.9375
40	0.88889	0.06519	2.84× 10${}^{-4}$	0.76113	1.01665	0.95111	0.0372	2.55× 10${}^{-5}$	0.87819	1.02403
50	0.89778	0.06478	2.05× 10${}^{-4}$	0.77082	1.02474	0.99111	0.01183	4.58× 10${}^{-6}$	0.96793	1.0143
60	0.96444	0.03088	1.46× 10${}^{-5}$	0.90391	1.02497	1	0	3.07× 10${}^{-6}$	1	1
70	0.99524	0.00787	5.65× 10${}^{-6}$	0.97981	1.01067	1	0	4.59× 10${}^{-6}$	1	1
80	1	0	1.13× 10${}^{-5}$	1	1	1	0	1.13× 10${}^{-5}$	1	1
90	1	0	1.13× 10${}^{-5}$	1	1	1	0	3.07× 10${}^{-6}$	1	1
	Inertia					Correlation				
P.S.($\phi_{s}$) %	AUC	Std. Error	Asymptotic Prob	95% LCL	95% UCL	AUC	Std. Error	Asymptotic Prob	95% LCL	95% UCL
10	0.69333	0.09833	0.07118	0.50061	0.88606	0.33778	0.10267	0.13004	0.13654	0.53901
20	0.51282	0.11935	0.90832	0.2789	0.74675	0.43846	0.11819	0.58041	0.20681	0.67011
30	0.6	0.10963	0.35069	0.38514	0.81486	0.82667	0.07914	0.0023	0.67155	0.98178
40	0.54222	0.10975	0.69355	0.32712	0.75732	0.83333	0.07762	0.00225	0.68121	0.98546
50	0.8	0.0797	0.00511	0.64378	0.95622	0.62667	0.10584	0.23716	0.41923	0.83411
60	0.79111	0.09878	0.00659	0.5975	0.98473	0.57778	0.10858	0.46792	0.36497	0.79059
70	0.88571	0.06707	4.08× 10${}^{-4}$	0.75425	1.01718	0.52381	0.11333	0.82726	0.30169	0.74593
80	0.81667	0.1075	0.00541	0.60598	1.02736	0.89722	0.07095	4.85× 10${}^{-4}$	0.75816	1.03628
90	0.76	0.10475	0.01525	0.55469	0.96531	1	0	3.07× 10${}^{-6}$	1	1
	IDM					Entropy				
P.S.($\phi_{s}$) %	AUC	Std. Error	Asymptotic Prob	95% LCL	95% UCL	AUC	Std. Error	Asymptotic Prob	95% LCL	95% UCL
10	0.53556	0.11144	0.74002	0.31714	0.75397	0.52444	0.11011	0.81955	0.30864	0.74025
20	0.63333	0.11334	0.23103	0.4112	0.85547	0.63333	0.11357	0.23103	0.41074	0.85593
30	0.77778	0.09229	0.00953	0.59689	0.95866	0.62889	0.10737	0.22903	0.41845	0.83933
40	0.91111	0.05535	1.25× 10${}^{-4}$	0.80262	1.0196	0.79778	0.0831	0.00545	0.63491	0.96064
50	0.97778	0.02425	8.24× 10${}^{-6}$	0.93024	1.02531	0.92444	0.04749	7.46× 10${}^{-5}$	0.83138	1.01751
60	1	0	3.07× 10${}^{-6}$	1	1	0.94	0.04441	4.02× 10${}^{-5}$	0.85296	1.02704
70	1	0	4.59× 10${}^{-6}$	1	1	0.96667	0.02964	1.89× 10${}^{-5}$	0.90858	1.02475
80	1	0	1.13× 10${}^{-5}$	1	1	1	0	1.13× 10${}^{-5}$	1	1
90	1	0	3.07× 10${}^{-6}$	1	1	1	0	3.07× 10${}^{-6}$	1	1
COMPLETE										
	Mean Pixel I.					Energy				
P.S. %	AUC	Std. Error	Asymptotic Prob	95% LCL	95% UCL	AUC	Std. Error	Asymptotic Prob	95% LCL	95% UCL
10	0.82	0.08209	0.00282	0.6591	0.9809	0.49556	0.10757	0.96691	0.28472	0.70639
20	0.72821	0.10339	0.04037	0.52557	0.93084	0.79231	0.08537	0.00865	0.62499	0.95963
30	0.57778	0.10681	0.46792	0.36843	0.78712	0.58889	0.10693	0.40679	0.37931	0.79847
40	0.72	0.09515	0.04006	0.53351	0.90649	0.74	0.08984	0.0251	0.56393	0.91607
50	0.92222	0.05083	8.13× 10${}^{-5}$	0.8226	1.02184	0.84	0.07163	0.00151	0.69962	0.98038
60	0.96667	0.03433	1.33× 10${}^{-5}$	0.89938	1.03396	0.56444	0.1095	0.54755	0.34982	0.77907
70	0.99048	0.01254	6.95× 10${}^{-5}$	0.9659	1.01505	0.7119	0.09701	0.05212	0.52177	0.90204
80	1	0	1.13× 10${}^{-5}$	1	1	0.56944	0.11426	0.5419	0.3455	0.79339
90	1	0	3.07× 10${}^{-6}$	1	1	0.68444	0.10063	0.08519	0.48721	0.88168
	Inertia					Correlation				
P.S.($\phi_{s}$) %	AUC	Std. Error	Asymptotic Prob	95% LCL	95% UCL	AUC	Std. Error	Asymptotic Prob	95% LCL	95% UCL
10	0.59556	0.1093	0.37251	0.38134	0.80977	0.70667	0.0984	0.05376	0.51381	0.89952
20	0.5641	0.12243	0.56474	0.32414	0.80407	0.56923	0.11945	0.53402	0.33512	0.80334
30	0.60889	0.10971	0.30953	0.39386	0.82392	0.62667	0.10653	0.23716	0.41787	0.83547
40	0.52444	0.10859	0.81955	0.31161	0.73728	0.82222	0.07846	0.00264	0.66844	0.976
50	0.74667	0.08949	0.02133	0.57127	0.92207	0.83333	0.0747	0.00187	0.68693	0.97974
60	0.75556	0.10351	0.01708	0.55267	0.95844	0.81333	0.0791	0.00345	0.65829	0.96837
70	0.84762	0.07623	0.00144	0.69821	0.99703	0.84048	0.07949	0.00181	0.68469	0.99627
80	0.73889	0.11734	0.03589	0.50891	0.96887	0.99444	0.00922	1.41× 10${}^{-5}$	0.97637	1.01252
90	0.68	0.11305	0.09298	0.45842	0.90158	1	0	3.07× 10${}^{-6}$	1	1
	IDM					Entropy				
P.S.($\phi_{s}$) %	AUC	Std. Error	Asymptotic Prob	95% LCL	95% UCL	AUC	Std. Error	Asymptotic Prob	95% LCL	95% UCL
10	0.50444	0.10811	0.96691	0.29255	0.71634	0.55333	0.11077	0.61867	0.33623	0.77044
20	0.76154	0.09494	0.01881	0.57545	0.94762	0.72821	0.10749	0.04037	0.51753	0.93888
30	0.50667	0.11263	0.95039	0.28591	0.72743	0.53778	0.11455	0.72442	0.31326	0.7623
40	0.68222	0.09928	0.08902	0.48764	0.87681	0.71111	0.09812	0.04881	0.5188	0.90342
50	0.86444	0.06482	6.71× 10${}^{-4}$	0.7374	0.99148	0.84889	0.07013	0.00113	0.71143	0.98635
60	0.79111	0.08757	0.00659	0.61947	0.96275	0.81556	0.09132	0.00323	0.63658	0.99453
70	0.92143	0.04884	1.12× 10${}^{-4}$	0.8257	1.01716	0.88571	0.06712	4.08× 10${}^{-4}$	0.75416	1.01726
80	0.86667	0.07464	0.00128	0.72038	1.01296	0.9	0.07463	4.43× 10${}^{-4}$	0.75372	1.04628
90	0.90444	0.05854	1.60× 10${}^{-4}$	0.78971	1.01918	0.85111	0.0797	0.00105	0.69489	1.00733

**Table 2:** Areas under ROC curve (AUC) for dilution detection of Clonazepam, Ciprofloxacin and Budesonide. Texture parameters were estimated for different regions of the stains: central region (entropy), full stain (entropy), and the central aggregated (area), respectively. The different regions were chosen based on the morphological characteristics of the deposits. Ps = water content (%). Error = standard error. Asym. prob.: asymptotic probability. LCL: the lower confidence limit. UCL: the upper confidence limit.

Clonazepam					
	Entropy				
P.S. ($\phi_{s}$) %	AUC	Std. Error	Asymptotic Prob	95% LCL	95% UCL
10	0.73016	0.13326	0.06773	0.46897	0.99135
20	0.74107	0.11309	0.06536	0.51941	0.96273
30	0.9881	0.01542	2.47 × 10${}^{-5}$	0.95787	1.01833
40	0.86364	0.07332	0.00217	0.71993	1.00734
50	0.91837	0.04996	1.65 × 10${}^{-4}$	0.82045	1.01628
Ciprofloxacin					
	Entropy				
P.S.($\phi_{s}$) %	AUC	Std. Error	Asymptotic Prob	95% LCL	95% UCL
10	0.99111	0.01183	4.58 × 10${}^{-6}$	0.96793	1.0143
20	1	0	7.08 × 10${}^{-6}$	1	1
30	1	0	1.13 × 10${}^{-5}$	1	1
40	1	0	7.08 × 10${}^{-6}$	1	1
50	1	0	2.10 × 10${}^{-6}$	1	1
Budesonide					
	Aggregate size				
P.S.($\phi_{s}$) %	AUC	Std. Error	Asymptotic Prob	95% LCL	95% UCL
10	0.72444	0.10247	0.0362	0.5236	0.92529
20	0.77778	0.09272	0.00953	0.59605	0.95951
30	0.95556	0.03616	2.12 × 10${}^{-5}$	0.88469	1.02642
40	1	0	3.07 × 10${}^{-6}$	1	1
50	1	0	3.07 × 10${}^{-6}$	1	1
